# Association of temporal and masseter muscle thickness with cognitive function and clinical severity in Alzheimer’s disease: a retrospective study

**DOI:** 10.3389/fnins.2026.1905634

**Published:** 2026-09-28

**Authors:** Jing Li, Xingqi Xiang, Siyu Long, Fengqiong Hu, Fu Ding

**Affiliations:** 1 Department of Nursing, The First Affiliated Hospital of Chongqing Medical University, Chongqing, China; 2 Day Service Center, The First Affiliated Hospital of Chongqing Medical University, Chongqing, China; 3 Department of Nephrology, South China Hospital of Shenzhen University, Shenzhen, China; 4 Department of Nuclear Medicine, The First Affiliated Hospital of Chongqing Medical University, Chongqing, China

**Keywords:** Alzheimer’s disease, biomarker, cognitive function, masseter muscle thickness, temporal muscle thickness

## Abstract

**Background:**

Temporal muscle thickness (TMT) has emerged as an imaging marker of skeletal muscle status and may be associated with cognitive decline in Alzheimer’s disease (AD). However, its relationship with AD severity remains unclear, and the clinical relevance of masseter muscle thickness (MMT) is unknown.

**Objective:**

To evaluate the associations of TMT and MMT with cognitive function and disease severity in AD.

**Methods:**

This retrospective study included 242 patients with AD between December 2023 and April 2025. TMT and MMT were measured on axial T1-weighted MRI, cognitive function was assessed using standardized neuropsychological tests, and disease severity was determined by Clinical Dementia Rating (CDR).

**Results:**

The intraclass correlation coefficients (ICC) for the mean TMT and MMT were 0.868 and 0.896, respectively, indicating good consistency. As the severity of AD progresses, TMT tends to be thinner in severe AD than in mild AD. Spearman’s correlation analysis revealed that both TMT and MMT were correlated with the Mini-Mental State Examination (MMSE) and CDR. After adjusting for various confounding factors using ordered logistic regression and multiple linear regression, TMT was found to be independently associated with AD severity (OR: 0.424, 95% CI: 0.290–0.618, *p* < 0.001) and positively correlated with MMSE scores. MMT showed only a weak independent association with CDR staging (OR: 0.830, 95% CI: 0.724–0.952, *p* = 0.008), while no significant correlation was observed with the MMSE. Stratified analysis revealed that the TMT maintained a consistent association in both male and female patients, whereas the MMT achieved statistical significance only in females in ordered logistic regression; all other findings were not statistically significant.

**Conclusion:**

Temporal muscle thickness is associated with cognitive impairment and clinical severity in patients with AD and may serve as a simple, noninvasive imaging marker; in contrast, the independent associations of MMT with disease severity were weak and sex-specific, suggesting that its clinical utility may be limited and requires further validation.

## 1 Introduction

Alzheimer’s disease is a neurodegenerative disorder characterized by progressive cognitive decline and behavioral abnormalities, primarily affecting older adults and individuals in the presenile stage (Jia et al., 2024). Its clinical manifestations include memory impairment, deficits in spatial orientation, language disorders, apraxia, executive dysfunction, and neuropsychiatric symptoms (Scheltens et al., 2021). AD is characterized by its high incidence, high mortality rate, and significant caregiving burden (Alzheimer’s Association, 2024). Therefore, early screening for AD risk is of great significance for the prevention and management of this serious condition. Studies have shown that reduced overall muscle mass is associated with an accelerated decline in cognitive function among older adults (Barone et al., 2024). Sarcopenia has been established as a risk factor for AD (Beeri et al., 2021), and multiple pathways may explain the comorbid mechanisms linking sarcopenia and AD (Yang et al., 2022; Ye et al., 2023; Brisendine and Drake, 2026). It is crucial to investigate biomarkers associated with sarcopenia to elucidate their relationship with AD and predict its onset. TMT serves as a valid surrogate marker for assessing skeletal muscle mass and can be used in the evaluation and diagnosis of sarcopenia (Sifil and Kahn, 2021; Yilmaz et al., 2025). It may also reflect muscle quality in patients with neurological conditions as a proxy for overall skeletal muscle status (Steindl et al., 2020; Cho et al., 2022). Studies have indicated that TMT exhibits a significant correlation with cognitive function and may even serve as a novel indicator for predicting the risk of dementia, offering a new approach for early detection (Cho et al., 2022; Borda et al., 2024; Zhu et al., 2025). However, existing studies have not clarified the association between TMT and AD severity. Furthermore, it remains unclear whether MMT, another major masticatory muscle, can also be correlated with cognitive function in patients with dementia (Salinas-Rodríguez et al., 2021). In summary, we hypothesized that (1) TMT would be associated with cognitive function and AD severity as classified by CDR; and (2) MMT would also be associated with cognitive function in patients with AD.

## 2 Materials and methods

### 2.1 Participants

This retrospective study included 242 patients with cognitive impairment. All patients with cognitive impairment were diagnosed via positron emission tomography computed tomography (PET-CT) at the First Affiliated Hospital of Chongqing Medical University between December 2023 and April 2025. Based on CDR scores of 1, 2, and 3, patients were categorized as follows: mild AD, 72 cases; moderate AD, 77 cases; and severe AD, 93 cases. The data filtering process is shown in Figure 1.

**FIGURE 1:**
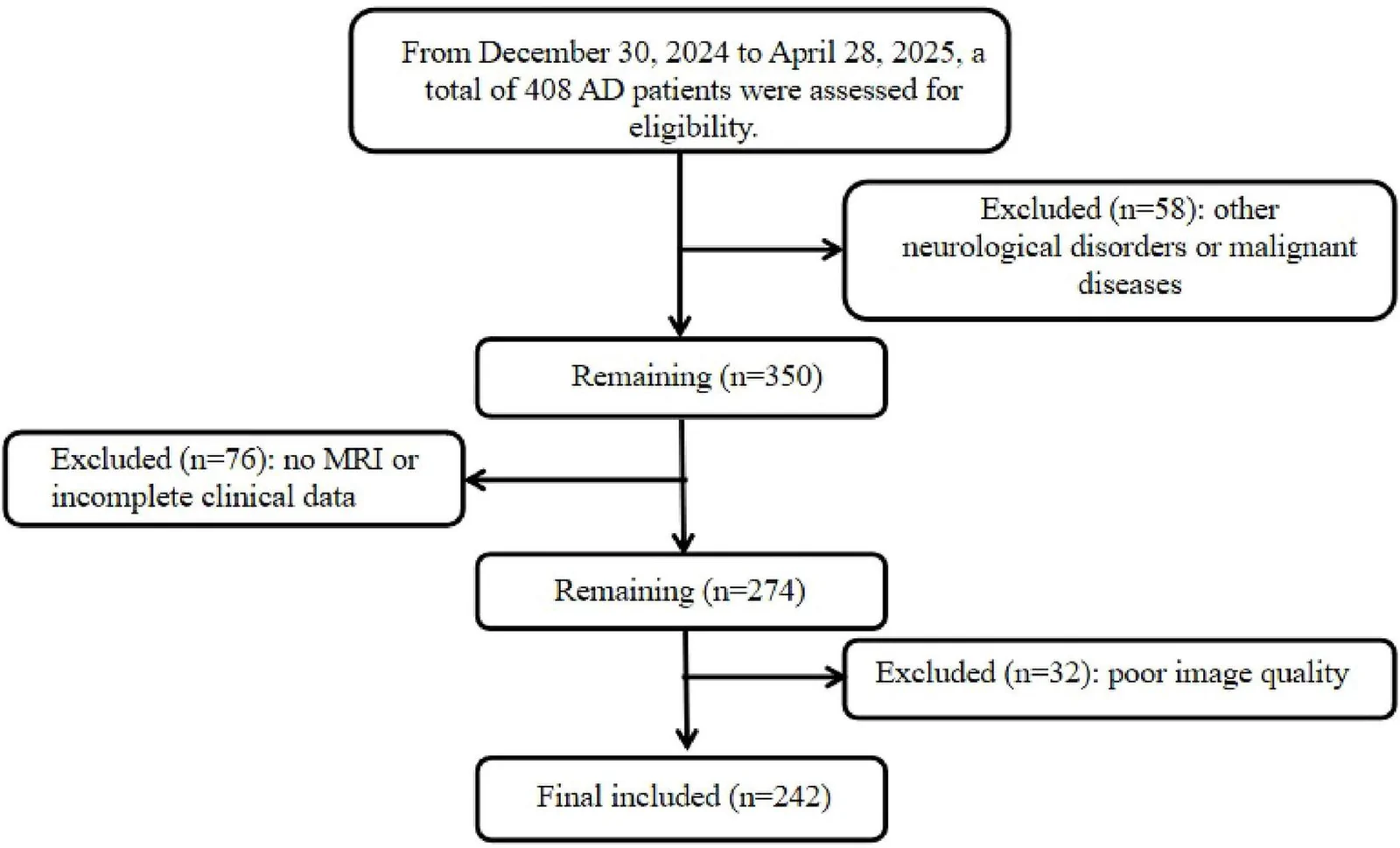
Flowchart of the participant screening process.

The inclusion criteria for patients were as follows: (1) age ≥ 60 years; (2) complete clinical records; and (3) confirmed by clinicians using PET-CT. The exclusion criteria were as follows: (1) severe hepatic, renal, cardiac, or pulmonary insufficiency; (2) presence of other psychiatric disorders; (3) any type of tumor or infectious disease; (4) other types of dementia (including frontotemporal dementia, Parkinson’s disease dementia, Lewy body dementia, and vascular dementia); (5) history of masticatory muscle surgery. (6) History of stroke or other cerebrovascular diseases. This retrospective study was conducted at the First Affiliated Hospital of Chongqing Medical University, Chongqing, China, and was approved by the local Ethics Committee (Approval No. 2025-643-01). The study was conducted in accordance with the Declaration of Helsinki. The raw data of this study involve patient privacy information and are not publicly available due to institutional ethical restrictions and patient consent agreements. All MRI images have been anonymized to remove identifiable facial features. De-identified data may be obtained from the corresponding author upon reasonable request and after approval from the ethics committee.

### 2.2 Clinical data and cognitive assessment

The clinical data collected from the electronic medical record system for this study included age, height, weight, BMI, history of chronic diseases, years of education, smoking and drinking history, presence of the APOE ε4 genotype, and scores on the cognitive assessment scale. Cognitive assessments were conducted by trained professionals using the MMSE and the CDR. According to the 2011 National Institute on Aging–Alzheimer’s Association (NIA-AA) criteria, the CDR score is officially used to classify AD dementia as mild, moderate, or severe (Albert et al., 2011). All PET-CT examinations were performed in the Department of Nuclear Medicine at the First Affiliated Hospital of Chongqing Medical University using a Philips PET/CT scanner. All patients were diagnosed with AD based on double-positive PET-CT results for ^18^F-AV45 (β-amyloid) and ^18^F-MK6240 (tau protein). Aβ positivity was defined as a cortical standardized uptake value ratio (SUVR) ≥ 1.11 (Landau et al., 2013). Tau positivity was defined as a SUVR ≥ 1.35 in the medial temporal lobe or neocortex (Leuzy et al., 2021). All PET-CT images were independently interpreted by two experienced nuclear medicine physicians. In cases of interpretation discrepancies, consensus was reached through joint discussion; after the nuclear medicine department submitted the report, clinicians made the final diagnosis based on the patient’s medical history and clinical features. Brain MRI and neuropsychological evaluations were performed within a 10-days interval. In this study, chronic disease, as listed in Table 1, is defined as the presence of at least one of the following medically diagnosed conditions: hypertension, diabetes, or coronary heart disease. Family history is defined as a positive history of dementia (including AD or other related dementias) among first- or second-degree relatives. APOE ε4 genotyping data were extracted from the patients’ electronic medical record system and performed by the hospital’s clinical laboratory. Individuals carrying at least one ε4 allele were classified as APOE ε4-positive.

**TABLE 1:** Demographic and clinical characteristics of participants by cognitive status.

Variable	Overall	Mild AD (*N* = 72)	Moderate AD (*N* = 77)	Severe AD (*N* = 93)	*P*
Age, years	73.00 (69.00, 78.00)	72.00 (68.00, 78.00)	74.00 (70.00, 79.00)	72.00 (66.00, 77.00)	0.280
Education, years	9.00 (6.00, 12.00)	9.00 (6.00, 12.00)	9.00 (6.00, 12.00)	9.00 (6.00, 12.00)	0.421
MMSE	6.00 (1.00, 13.00)	18.00 (13.00, 21.00)^∧^a	7.00 (5.00, 11.00)^∧^b	0.00 (0.00, 3.00)^∧^c	<0.001
BMI, kg/m^2^	22.64 ± 2.87	22.71 ± 2.83	22.20 ± 2.52	22.94 ± 3.16	0.235
TMT, mm	7.56 (7.04, 8.12)	7.79 (7.43, 8.25)^∧^a	7.60 (6.78, 8.12)^∧^a,b	7.33 (6.96, 7.80)^∧^b	<0.001
MMT, mm	12.99 ± 1.81	13.41 ± 1.85	12.90 ± 2.03	12.74 ± 1.54	0.053
ASM, kg	14.68 (12.59, 18.61)	14.46 (12.66, 19.69)	14.70 (12.50, 18.61)	14.77 (12.63, 17.59)	0.908
ASMI, kg/m^2^	6.02 (5.36, 7.15)	6.09 (5.26, 7.23)	5.94 (5.35, 7.05)	6.07 (5.42, 7.02)	0.921
Sex					0.848
Male	82 (33.88%)	24 (33.33%)	28 (36.36%)	30 (32.26%)	
Female	160 (66.12%)	48 (66.67%)	49 (63.64%)	63 (67.74%)	
Smoking					0.042
No	199 (82.23%)	66 (91.67%)^∧^a	61 (79.22%)^∧^a	72 (77.42%)^∧^a	
Yes	43 (17.77%)	6 (8.33%)^∧^a	16 (20.78%)^∧^a	21 (22.58%)^∧^a	
Alcohol consumption					0.738
No	195 (80.58%)	60 (83.33%)	62 (80.52%)	73 (78.49%)	
Yes	47 (19.42%)	12 (16.67%)	15 (19.48%)	20 (21.51%)	
Hypertension					0.913
No	167 (69.01%)	51 (70.83%)	53 (68.83%)	63 (67.74%)	
Yes	75 (30.99%)	21 (29.17%)	24 (31.17%)	30 (32.26%)	
Diabetes mellitus					0.893
No	213 (88.02%)	63 (87.50%)	67 (87.01%)	83 (89.25%)	
Yes	29 (11.98%)	9 (12.50%)	10 (12.99%)	10 (10.75%)	
Coronary heart disease					0.038
No	224 (92.56%)	69 (95.83%)^∧^a	74 (96.10%)^∧^a	81 (87.10%)^∧^a	
Yes	18 (7.44%)	3 (4.17%)^∧^a	3 (3.90%)^∧^a	12 (12.90%)^∧^a	
Chronic disease					0.236
No	148 (61.16%)	40 (55.56%)	45 (58.44%)	63 (67.74%)	
Yes	94 (38.84%)	32 (44.44%)	32 (41.56%)	30 (32.26%)	
Family history					0.362
No	188 (77.69%)	52 (72.22%)	63 (81.82%)	73 (78.49%)	
Yes	54 (22.31%)	20 (27.78%)	14 (18.18%)	20 (21.51%)	
APOE ε4 positive					0.527
No	150 (61.98%)	48 (66.67%)	48 (62.34%)	54 (58.06%)	
Yes	92 (38.02%)	24 (33.33%)	29 (37.66%)	39 (41.94%)	

Data are presented as mean ± standard deviation for normally distributed continuous variables, as median (interquartile range) for non-normally distributed continuous variables, and as *n* (%) for categorical variables. Variables with a normal distribution were compared using analysis of variance (ANOVA), while variables with a non-normal distribution were compared using the Kruskal-Wallis test. Chi-square test for categorical variables. Pairwise comparisons were adjusted using the Bonferroni correction. Within each row, values sharing the same superscript letter are not significantly different after Bonferroni correction (adjusted *P* > 0.05). Smoking and alcohol consumption were treated as binary variables, defined as “Yes” (indicating a history of past or current smoking or alcohol consumption) and “No” (indicating no lifetime exposure).

### 2.3 TMT and MMT measurement

Cranial magnetic resonance imaging (MRI) examinations were performed using a MAGNETOM ESSENZA 1.5T medical MRI scanner manufactured by Siemens of Germany. Measurements were taken on uncontrast-enhanced or contrast-enhanced T1-weighted images (T1WI) of the brain (slice thickness 0.8 mm, slice spacing 0 mm), and the TMT and MMT were measured using the basic measurement tools of the PACS system. Representative TMT and MMT measurements are illustrated in Figures 2, 3, respectively. On the axial MRI slice passing through the root of the nose, identify the deepest point of the temporal bone on that slice and measure the thickness of the temporalis muscle (excluding subcutaneous fat and skin) along a direction perpendicular to the long axis of the muscle (Chen et al., 2023). On the axial slice where the mandibular ramus is most clearly visible, select the thickest point of the masseter muscle at the midpoint of the mandibular ramus for measurement (Atasoy et al., 2025). Both independent measurers had undergone systematic training; under the supervision of a radiologist, they performed blinded measurements of TMT and MMT for each image. Bilateral TMT measurements were obtained for each patient, and the average of the left and right TMT values was calculated; this average was used in all subsequent analyses. The same approach was applied to MMT measurements: bilateral masseter muscle thickness was measured, and the mean of the left and right values was used for all subsequent analyses.

**FIGURE 2:**
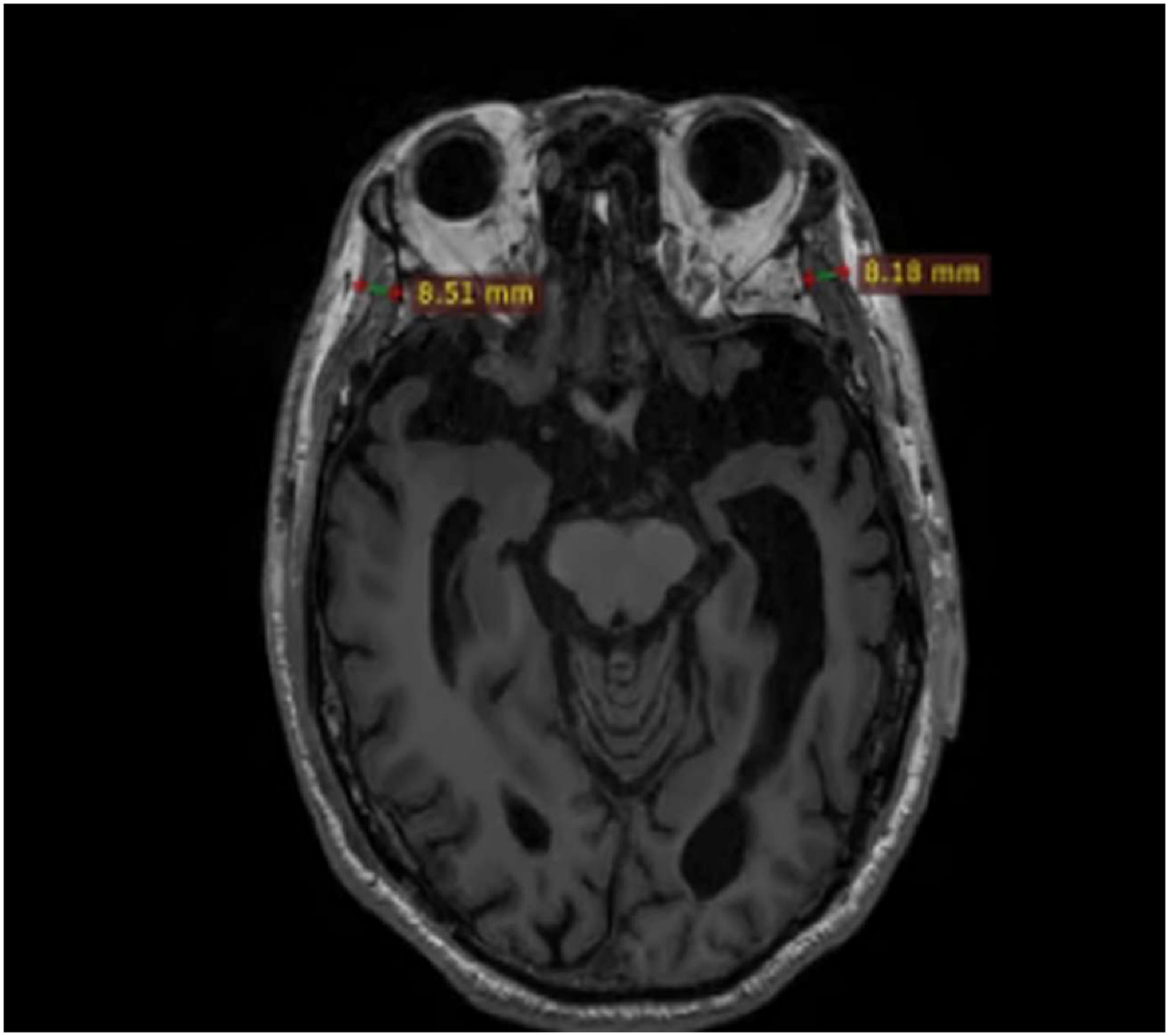
On the axial MRI slice passing through the root of the nose, identify the deepest point of the temporal bone on that slice and measure the thickness of the temporalis muscle (excluding subcutaneous fat and skin) along a direction perpendicular to the long axis of the muscle.

**FIGURE 3:**
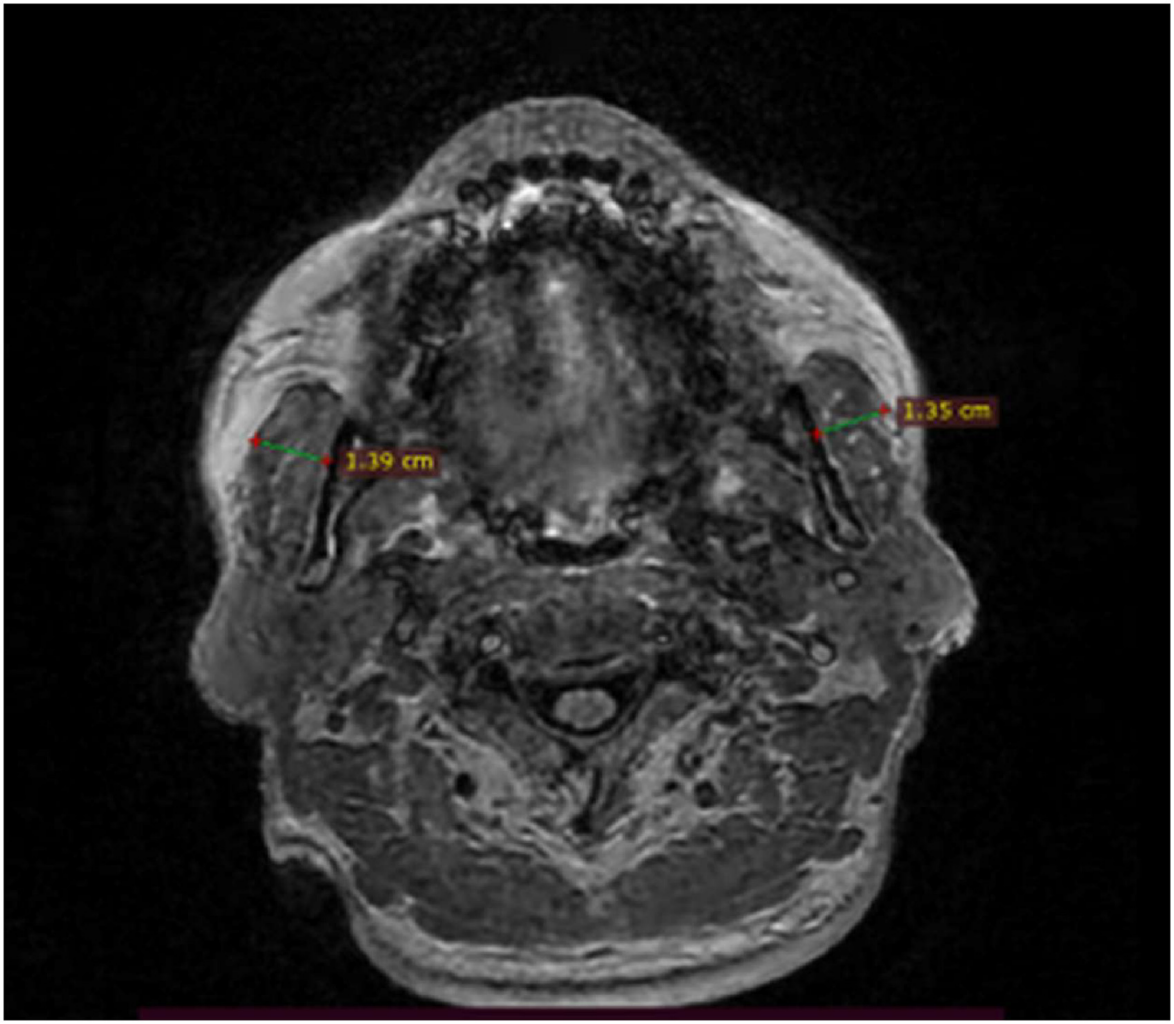
On the axial slice where the mandibular ramus is most clearly visible, select the thickest point of the masseter muscle at the midpoint of the mandibular ramus for measurement.

### 2.4 ASMI estimation

Previous studies have confirmed that sarcopenia is a risk factor for AD. Given that TMT serves as an effective surrogate marker for assessing skeletal muscle mass, this study conducted an exploratory analysis of the association between TMT and estimated skeletal muscle markers in patients with AD. In this study, the appendicular skeletal muscle index (ASMI) was calculated by dividing appendicular skeletal muscle mass (ASM) by height squared (m^2^). ASM was estimated using a validated anthropometric equation for the Chinese population (Wen et al., 2011): ASM = 0.193 × body weight (kg) + 0.107 × height (cm) − 4.157 × sex (1 for males, 2 for females) − 0.037 × age (years) − 2.631.

### 2.5 Statistical analysis

Statistical analyses were performed using R (version 4.3.3). Continuous variables were first tested for normality using the Shapiro-Wilk test. Variables following a normal distribution were expressed as mean ± standard deviation, and between-group comparisons were performed using analysis of variance (ANOVA). Variables not following a normal distribution were expressed as median and interquartile range, and between-group comparisons were performed using the Kruskal-Wallis rank-sum test. Categorical variables were expressed as counts and frequencies, and between-group comparisons were performed using the chi-square test. Inter-rater reliability for the TMT and MMT was assessed using the ICC, based on a two-way mixed absolute agreement model. Since all subsequent analyses used the average of the left- and right-side scores, the ICC reported is for the average score. Point estimates and 95% confidence intervals are reported; An ICC < 0.50 is considered poor, 0.50–0.75 is moderate, 0.75–0.90 is good, and >0.90 is excellent (Koo and Li, 2016). During the evaluation process, both independent raters were blinded to all patients’ clinical information, including cognitive function scores, demographic data, and other covariates. *Post hoc* pairwise comparisons were conducted using the Bonferroni correction. Spearman’s correlation analysis was used to examine the correlations among the variables. Ordinal logistic regression and multiple linear regression models were employed to evaluate the associations between each variable and the categorical outcome variables and continuous variables, respectively, under different covariate control strategies. All statistical analyses were two-sided, and a *P*-value of <0.05 was considered statistically significant. For ordinal logistic regression models, the Brant test was used to evaluate the proportional odds hypothesis. Model fit was compared using the Akaike Information Criterion (AIC). For the multiple linear regression model with MMSE as the dependent variable, residual diagnostics were performed using scatter plots of residuals versus fitted values, normal Q-Q plots, scale-location plots, and scatter plots of residuals versus leverage values. To address potential heteroscedasticity, HC3 heteroscedasticity-consistent standard errors were calculated as part of a sensitivity analysis. All supplementary diagnostic tables and figures are provided in the online Supplementary material.

## 3 Results

### 3.1 Characteristics of enrolled participants

This study included a total of 242 patients with PET-confirmed AD, comprising 82 men and 160 women. Based on the CDR scale, the participants were divided into a mild AD group (72 cases, 29.76%), a moderate AD group (77 cases, 31.82%), and a severe AD group (93 cases, 38.42%). The median age of all participants was 73.00 (69.00, 78.00) years, and the median temporal muscle thickness (TMT) was 7.56 (7.04, 8.12) mm. Results from the Kruskal–Wallis rank-sum test showed a statistically significant difference in TMT among the three groups (*P* < 0.001). After Bonferroni correction, MMSE remained significant in all pairwise comparisons, and TMT was significant only between severe and mild groups (adjusted *P* < 0.05); smoking and coronary heart disease showed no significant pairwise differences. Detailed demographic, clinical, and radiological data for all three groups are presented in Table 1. Detailed normality test results for all continuous variables are provided in Supplementary Table 1.

### 3.2 Reliability assessment of TMT and MMT

The ICCs calculated for each TMT and MMT measurement reflected high inter-rater reproducibility: the average-measure ICCs were 0.868 (95% CI: 0.830–0.898) for TMT and 0.896 (95% CI: 0.866–0.919) for MMT.

### 3.3 Associations of TMT with MMT, age, sex, ASMI, and cognitive function

Temporal muscle thickness was positively correlated with MMT (*r* = 0.356, *p* < 0.001) and MMSE scores (*r* = 0.266, *p* < 0.01), and negatively correlated with CDR scores (*r* = −0.263, *p* < 0.01). Additionally, TMT showed a significant positive correlation with ASMI (*r* = 0.404, *p* < 0.001). Both TMT and MMT are associated with sex, but TMT is not associated with age. Similarly, MMT was positively correlated with MMSE scores (*r* = 0.132, *p* = 0.040) and negatively correlated with CDR scores (*r* = −0.136, *p* = 0.035). The results of the Spearman correlation analysis between the indicators are shown in Figure 4 and Table 2.

**FIGURE 4:**
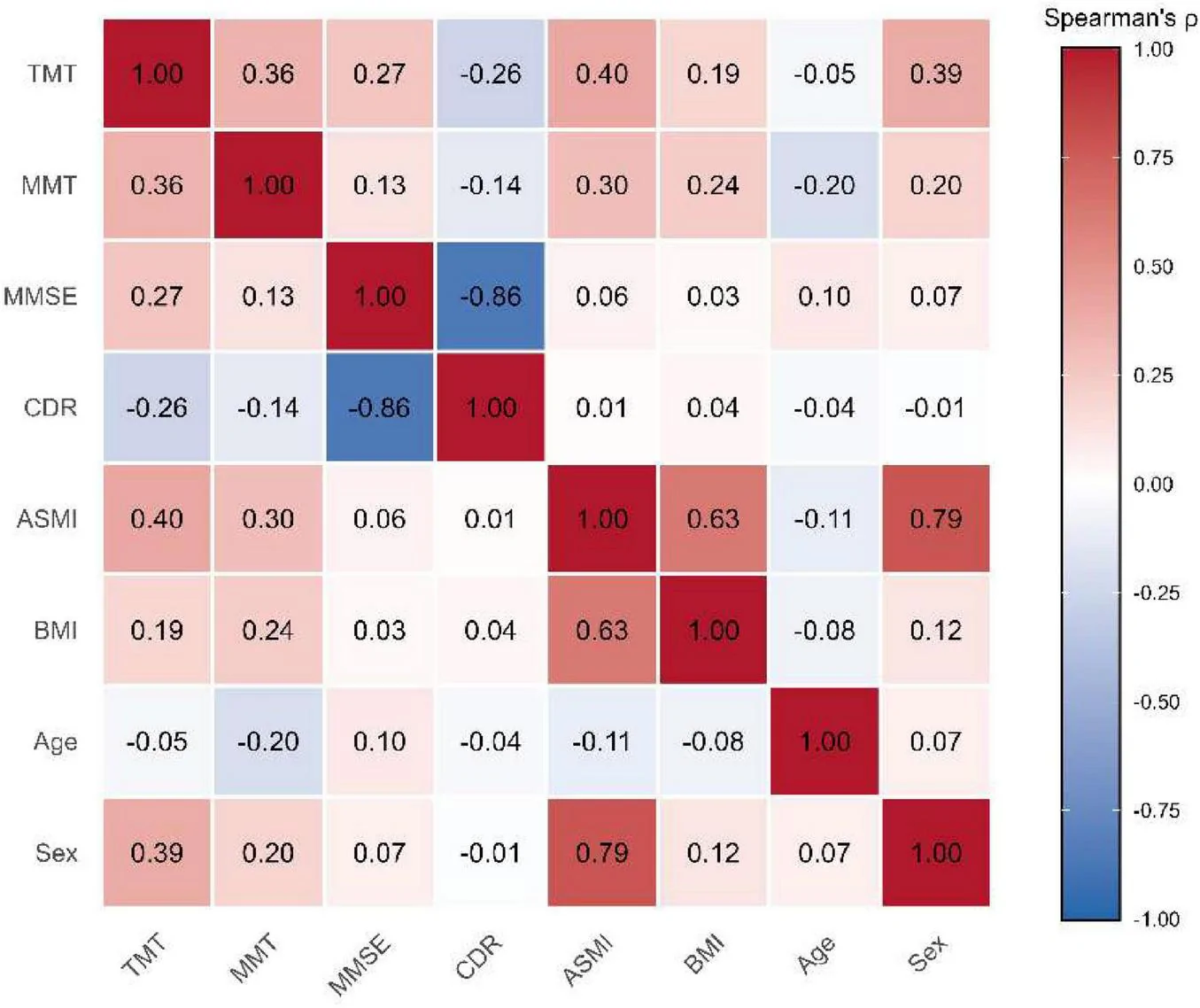
Correlation matrix of TMT, MMT, and clinical parameters in patients with AD.

**TABLE 2:** Analysis of correlations among MMSE, TMT, MMT, ASMI, BMI, and sex in patients with AD.

Variable	TMT	MMT	MMSE	CDR	ASMI	BMI	Age	Sex
TMT	1.000	0.356 (<0.001)	0.265 (<0.001)	–0.263 (<0.001)	0.404 (<0.001)	0.187 (0.003)	–0.048 (0.459)	0.387 (<0.001)
MMT	0.356 (<0.001)	1.000	0.132 (0.040)	–0.136 (0.035)	0.300 (<0.001)	0.236 (<0.001)	–0.202 (0.002)	0.200 (0.002)
MMSE	0.265 (<0.001)	0.132 (0.040)	1.000	–0.860 (<0.001)	0.060 (0.350)	0.034 (0.601)	0.102 (0.114)	0.071 (0.272)
CDR	–0.263 (<0.001)	–0.136 (0.035)	–0.860 (<0.001)	1.000	0.015 (0.817)	0.036 (0.577)	–0.036 (0.579)	–0.013 (0.839)
ASMI	0.404 (<0.001)	0.300 (<0.001)	0.060 (0.350)	0.015 (0.817)	1.000	0.628 (<0.001)	–0.109 (0.090)	0.786 (<0.001)
BMI	0.187 (0.003)	0.300 (<0.001)	0.034 (0.601)	0.036 (0.577)	0.628 (<0.001)	1.000	–0.078 (0.225)	0.121 (0.059)
Age	–0.048 (0.459)	–0.202 (0.002)	0.102 (0.114)	–0.036 (0.579)	–0.109 (0.090)	–0.078 (0.225)	1.000	0.068 (0.290)
Sex	0.387 (<0.001)	0.200 (0.002)	0.071 (0.272)	–0.013 (0.839)	0.786 (<0.001)	0.121 (0.059)	0.068 (0.290)	1.000

### 3.4 Ordered logistic regression analysis of TMT, MMT, and AD severity

This study employed a three-stage covariate adjustment strategy to construct an ordered logistic regression model, with the severity of AD, as measured by the CDR staging system, serving as the ordered dependent variable. The results of each model are detailed in Table 3. In all models, TMT was independently and negatively associated with AD severity, both in unadjusted analyses and after stepwise covariate adjustment. In model 3, which included age, sex, education years, family history, APOE ε4 genotype, and estimated appendicular skeletal muscle index (ASMI), the odds ratio (OR) for TMT per 1 mm increase was 0.424 (95% CI: 0.290–0.618, *P* < 0.001), suggesting that greater TMT was associated with lower AD severity. In contrast, MMT showed only a weak independent association in model 3 (OR: 0.830, 95% CI: 0.724–0.952, *P* = 0.008).

**TABLE 3:** Ordered logistic regression analysis of TMT, MMT, and AD severity under different adjustment strategies.

Exposure model	Model	Variable	β	OR (95% CI)	*P*
TMT	Model 1	TMT, per 1 mm	−0.647	0.524 (0.376, 0.729)	<0.001
	Model 2	TMT, per 1 mm	−0.793	0.452 (0.313, 0.654)	<0.001
		Age, years	−0.023	0.977 (0.943, 1.012)	0.194
		Male sex	0.488	1.629 (0.938, 2.831)	0.083
	Model 3	TMT, per 1 mm	−0.859	0.424 (0.290, 0.618)	<0.001
		Age, years	−0.018	0.982 (0.947, 1.018)	0.330
		Male sex	−0.003	0.997 (0.393, 2.529)	0.995
		Education, years	0.003	1.003 (0.948, 1.060)	0.928
		Family history, yes	−0.327	0.721 (0.404, 1.286)	0.268
		APOE ε4 positive	0.377	1.458 (0.884, 2.405)	0.140
		ASMI, kg/m^2^	0.303	1.354 (0.890, 2.059)	0.157
MMT	Model 1	MMT, per 1 mm	−0.143	0.867 (0.764, 0.983)	0.026
	Model 2	MMT, per 1 mm	−0.169	0.845 (0.739, 0.965)	0.013
		Age, years	−0.025	0.975 (0.941, 1.011)	0.166
		Male sex	0.130	1.139 (0.685, 1.895)	0.616
	Model 3	MMT, per 1 mm	−0.187	0.830 (0.724, 0.952)	0.008
		Age, years	−0.022	0.978 (0.943, 1.015)	0.238
		Male sex	−0.261	0.770 (0.308, 1.929)	0.577
		Education, years	−0.003	0.997 (0.943, 1.054)	0.908
		Family history, yes	−0.282	0.754 (0.424, 1.341)	0.337
		APOE ε4 positive	0.330	1.391 (0.849, 2.277)	0.190
		ASMI, kg/m^2^	0.242	1.273 (0.839, 1.934)	0.257

Model 1: unadjusted. Model 2: adjusted for age and sex. Model 3: adjusted for age, sex, education years, family history, APOE ε4 genotype and ASMI. Female sex, no family history, and APOE ε4 negative status were used as reference categories.

To further validate the stability of these associations, we performed stratified ordered logistic regression analyses by sex. After full adjustment for covariates (age, education years, family history, APOE ε4 genotype, and ASMI), TMT remained significantly associated with AD severity in both female and male subgroups. In contrast, MMT showed a statistically significant association only in the female subgroup, with no significant association observed in the male subgroup, suggesting marked sex heterogeneity in the relationship between MMT and AD severity. Detailed results for each model are presented in Table 4.

**TABLE 4:** Sensitivity analysis using ordered logistic regression for TMT, MMT, and AD severity, stratified by sex.

Sex subgroup	Model	Exposure	β	OR (95% CI)	*P*
Female	Model 1	TMT, per 1 mm	−0.647	0.523 (0.348, 0.788)	0.002
	Model 2	TMT, per 1 mm	−0.689	0.502 (0.331, 0.761)	0.001
	Model 3	TMT, per 1 mm	−0.753	0.471 (0.307, 0.722)	<0.001
	Model 1	MMT, per 1 mm	−0.165	0.848 (0.723, 0.994)	0.042
	Model 2	MMT, per 1 mm	−0.182	0.833 (0.707, 0.982)	0.029
	Model 3	MMT, per 1 mm	−0.215	0.806 (0.680, 0.956)	0.013
Male	Model 1	TMT, per 1 mm	−1.200	0.301 (0.132, 0.687)	0.004
	Model 2	TMT, per 1 mm	−1.190	0.304 (0.133, 0.697)	0.005
	Model 3	TMT, per 1 mm	−1.220	0.295 (0.125, 0.697)	0.005
	Model 1	MMT, per 1 mm	−0.111	0.895 (0.719, 1.114)	0.320
	Model 2	MMT, per 1 mm	−0.144	0.866 (0.690, 1.088)	0.217
	Model 3	MMT, per 1 mm	−0.137	0.872 (0.691, 1.101)	0.251

Model 1: unadjusted. Model 2: adjusted for age. Model 3: adjusted for age, education years, family history, APOE ε4 genotype and ASMI. No family history, and APOE ε4 negative status were used as reference categories.

Based on the results of the Brant test, none of the six ordinal logistic regression models violated the proportional odds assumption (all *P* > 0.05). The model fit statistics (AIC) are shown in Supplementary Table 2.

### 3.5 Multiple linear regression analysis of TMT, MMT, and MMSE scores

In this study, MMSE scores were used as a continuous cognitive outcome variable. Three multiple linear regression models with sequential covariate adjustment were constructed (Model 1: unadjusted; Model 2: adjusted for age and sex; Model 3: further adjusted for education years, family history, APOE ε4 genotype, and ASMI). The regression results are presented in Table 5. In Model 3, TMT showed a significant positive association with MMSE scores (β = 3.074, 95% CI: 1.606–4.541, *P* < 0.001), indicating that greater TMT was associated with higher cognitive scores. In contrast, MMT showed no statistically significant independent association with MMSE scores in model 3 (β = 0.505, 95% CI: −0.070 to 1.080, *P* = 0.085).

**TABLE 5:** Multiple linear regression analysis of TMT, MMT, and MMSE scores under different correction strategies.

Outcome	Model	Variable	β	β 95% CI	*P*
TMT	Model 1	TMT, per 1 mm	2.815	1.485, 4.146	<0.001
	Model 2	TMT, per 1 mm	3.110	1.660, 4.559	<0.001
		Age, years	0.117	−0.023, 0.257	0.101
		Male sex	−0.925	−3.131, 1.282	0.410
	Model 3	TMT, per 1 mm	3.074	1.606, 4.541	<0.001
		Age, years	0.123	−0.021, 0.268	0.094
		Male sex	−1.375	−5.073, 2.323	0.465
		Education, years	0.215	−0.020, 0.449	0.073
		Family history, yes	1.317	−0.981, 3.616	0.260
		APOE ε4 positive	−1.358	−3.365, 0.649	0.184
		ASMI, kg/m^2^	−0.045	−1.706, 1.616	0.957
MMT	Model 1	MMT, per 1 mm	0.492	−0.052, 1.036	0.076
	Model 2	MMT, per 1 mm	0.547	−0.019, 1.114	0.058
		Age, years	0.119	−0.028, 0.265	0.112
		Male sex	0.500	−1.637, 2.637	0.645
	Model 3	MMT, per 1 mm	0.505	−0.070, 1.080	0.085
		Age, years	0.131	−0.020, 0.281	0.088
		Male sex	−0.524	−4.317, 3.269	0.786
		Education, years	0.235	−0.006, 0.477	0.056
		Family history, yes	1.132	−1.231, 3.496	0.346
		APOE ε4 positive	−1.283	−3.348, 0.783	0.222
		ASMI, kg/m^2^	0.252	−1.465, 1.969	0.773

Model 1: unadjusted. Model 2: adjusted for age and sex. Model 3: adjusted for age, sex, education years, family history, APOE ε4 genotype and ASMI. Female sex, no family history, and APOE ε4 negative status were used as reference categories.

The results of the sensitivity analysis stratified by sex were consistent with the trends observed in the overall population: after full adjustment for age, education years, family history, APOE ε4 genotype, and ASMI, TMT remained independently and positively associated with MMSE scores in both male and female subgroups. In contrast, MMT showed no significant association with MMSE scores in either subgroup (Table 6).

**TABLE 6:** Sensitivity analysis of multiple linear regression for TMT, MMT, and MMSE scores after stratification by sex.

Sex subgroup	Model	Exposure	β	95% CI	*P*
Female	Model 1	TMT, per 1 mm	2.529	0.845, 4.213	0.003
	Model 2	TMT, per 1 mm	2.717	1.017, 4.418	0.002
	Model 3	TMT, per 1 mm	2.841	1.130, 4.551	0.001
	Model 1	MMT, per 1 mm	0.475	−0.203, 1.153	0.169
	Model 2	MMT, per 1 mm	0.554	−0.137, 1.244	0.115
	Model 3	MMT, per 1 mm	0.587	−0.116, 1.290	0.101
Male	Model 1	TMT, per 1 mm	4.413	1.514, 7.313	0.003
	Model 2	TMT, per 1 mm	4.349	1.436, 7.262	0.004
	Model 3	TMT, per 1 mm	3.832	0.706, 6.957	0.017
	Model 1	MMT, per 1 mm	0.410	−0.583, 1.403	0.414
	Model 2	MMT, per 1 mm	0.534	−0.483, 1.552	0.299
	Model 3	MMT, per 1 mm	0.330	−0.718, 1.377	0.533

Model 1: unadjusted. Model 2: adjusted for age. Model 3: adjusted for age, education years, family history, APOE ε4 genotype and ASMI. No family history, and APOE ε4 negative status were used as reference categories.

Residual diagnostics for the linear regression model and sensitivity analyses using HC3 robust standard errors did not substantially alter the significance or direction of the study results (Supplementary Figures 1, 2 and Supplementary Table 3).

## 4 Discussion

The TMT measurements observed in this study demonstrated high inter-rater reliability, confirming the feasibility of the TMT as a practical clinical indicator. The present findings are in line with previous studies and further expand the current evidence. TMT has been shown to serve as an intracranial surrogate marker for skeletal muscle status in neurological disorders (Katsuki et al., 2021; Yilmaz et al., 2025); it can predict disease onset and prognosis (Atasoy et al., 2025). Furthermore, a standardized TMT measurement protocol ensures comparability across different imaging modalities and clinical settings (İnce and Özçakar, 2025). Among 242 patients with AD, both the TMT and MMT showed significant associations with cognitive function and AD severity, and both tests demonstrated high measurement reliability. As the disease progressed, the TMT showed marked thinning and was independently associated with CDR staging and MMSE scores across all sexes. Although MMT showed a weak univariate association with measures of cognitive function and disease severity, its independent association with CDR staging was weak (OR = 0.830 in model 3) and was limited to women, a finding that may be partly attributable to the smaller male subgroup limiting statistical power; regardless of sex, it showed no independent association with MMSE scores. A key advantage of TMT measurement lies in its simplicity and cost-effectiveness. This metric can be derived directly from patients’ routine cranial MRI images without the need for additional tests, making it particularly suitable for rapid initial screening of clinically high-risk populations (Steindl et al., 2020; Ten Cate et al., 2022). Compared to the MMT, the TMT demonstrates consistent correlations across patients of both sexes and exhibits high measurement reproducibility. This makes it a non-invasive, convenient imaging surrogate marker with greater clinical utility in assessing the severity of AD.

A previous study by Cho et al. (2022) preliminarily revealed that TMT in patients with dementia is associated with ASMI and cognitive function. The correlation coefficients between cognitive function and TMT in patients with AD in this study are consistent with those findings, indicating that in patients with AD, TMT exhibits a comparable degree of association with cognitive function as represented by the MMSE scales. Zhu et al.’s (2025) research further confirmed the association between TMT and AD pathological features as well as overall cognitive ability in the elderly dementia patient population, supporting the view that TMT serves as a potential biomarker for neurodegenerative diseases. Previous studies have focused solely on the association between TMT and cognitive function; building on this, the present study further confirms that TMT can distinguish the clinical severity of AD. Results from multiple linear regression analysis, adjusted for all confounding factors, show that thicker TMT is associated with higher MMSE scores, confirming its independent correlational link with concurrent cognitive performance. By combining the MMSE and CDR cognitive function scales, this study confirms a cross-sectional association between TMT and the severity staging of AD, thereby expanding its clinical utility in disease staging.

Concurrently, studies have indicated that masseter muscle volume, a key component of the oral cavity, also correlates with cognitive function in elderly patients with AD (Özsürekci et al., 2022; Lin et al., 2023). This study also observed a correlation between the MMT and cognitive function; however, after adjusting for confounding variables, MMT showed no independent association with continuous MMSE scores after adjustment. Only in the CDR staging model was a weak association observed between the MMT and AD severity, and this effect was observed exclusively in female patients; among male patients, the sample size was relatively small and insufficient to detect weaker associations. The significant sex difference may be partly explained by women’s higher risk of AD (O’Neal, 2024) and their more pronounced age-related decline in masticatory function (González-Fernández et al., 2021), though this finding warrants further investigation. However, it should be noted that the association between the TMT and cognitive function identified in this study does not, in and of itself, equate to incremental clinical value beyond that provided by standard cognitive assessments. Whether the TMT can offer additional value for risk stratification or decision-making in clinical practice requires prospective studies that directly compare its predictive performance with that of existing tools. Therefore, our findings should be viewed as proposing a hypothesis and supporting further research, rather than as evidence of clinical utility.

Temporal muscle thickness and MMT, as surrogate markers for total skeletal muscle mass, could be influenced by chewing limitations secondary to tooth loss or periodontal disease (Yi et al., 2025), which may contribute to masticatory muscle atrophy and, consequently, affect nutritional status, physical activity, and metabolic health. These factors have been linked to cognitive reserve and resilience against neuropathological damage (Chou et al., 2023). The association between TMT and cognitive function is plausibly mediated by several pathways connecting sarcopenia to cognitive impairment, including systemic inflammation, insulin resistance, and alterations in neurotrophic factor levels (Song et al., 2023; Arosio and Picca, 2026; Borda et al., 2026), as well as the potential effects of masticatory function on cerebral blood flow and neural activity. Reduced cerebral blood flow has been associated with elevated glutamate and decreased hippocampal pyramidal cell counts, which may contribute to cognitive decline (Zhang et al., 2024). As a surrogate for skeletal muscle mass, TMT may also indirectly reflect broader aspects of systemic health, such as nutrition and metabolic status (Salazar-Londoño et al., 2025). These mechanistic hypotheses, however, are speculative and await confirmation through basic research and longitudinal studies.

This study has the following limitations: First, as a retrospective study, it lacks data on dual-energy X-ray absorptiometry (DXA), oral health, and nutritional status; therefore, it is unable to account for the confounding effects of oral diseases on TMT and MMT. Furthermore, ASMI can only be estimated using a formula, and the calculated results may be subject to some bias; it is used solely as an exploratory indicator. Future studies will need to incorporate direct measurements of ASMI, such as DXA, to validate the findings. Second, it was not possible to confirm that TMT atrophy precedes the onset of cognitive symptoms; future longitudinal cohort studies are needed to determine its diagnostic cutoff. Third, the sample sizes for men and women were uneven, and the conclusions regarding the predictive value of the MMT for cognitive function in AD patients require validation in larger samples.

## 5 Conclusion

In this study, TMT measured by cranial MRI was independently associated with cognitive function and disease severity in patients with AD, suggesting its potential as a simple and noninvasive imaging biomarker. In contrast, MMT showed only a weak and sex-specific independent association, suggesting that its clinical utility in this context may be limited and that further research is needed in larger, more balanced cohorts.

## Data Availability

The raw data supporting the conclusions of this article will be made available by the authors, without undue reservation.
