# Supplementary material for: Association of temporal and masseter muscle thickness with cognitive function and clinical severity in Alzheimer’s disease: a retrospective study
Source: Front Neurosci. 2026 Sep 28;20:1905634. doi: 10.3389/fnins.2026.1905634 (PMC13655124; doi:10.3389/fnins.2026.1905634)
Supplement: Supplementary file 1 [file Data_Sheet_1.pdf]

Supplementary Table 1. Normality test results (Shapiro-Wilk test) for continuous variables in the study

**Variable**	**Overall**	**Mild AD (N = 72)**	**Moderate AD (N = 77)**	**Severe AD (N = 93)**
Age, years	W=0.975, P<0.001	W=0.971, P=0.097	W=0.970, P=0.068	W=0.960, P=0.006
Education, years	W=0.917, P<0.001	W=0.906, P<0.001	W=0.904, P<0.001	W=0.909, P<0.001
MMSE	W=0.890, P<0.001	W=0.976, P=0.183	W=0.952, P=0.005	W=0.781, P<0.001
BMI, kg/m²	W=0.991, P=0.150	W=0.983, P=0.445	W=0.984, P=0.467	W=0.985, P=0.350
TMT, mm	W=0.978, P<0.001	W=0.980, P=0.295	W=0.933, P<0.001	W=0.986, P=0.425
MMT, mm	W=0.990, P=0.092	W=0.985, P=0.532	W=0.980, P=0.257	W=0.978, P=0.123
ASM, kg	W=0.929, P<0.001	W=0.903, P<0.001	W=0.940, P=0.001	W=0.921, P<0.001
ASMI, kg/m²	W=0.959, P<0.001	W=0.956, P=0.012	W=0.944, P=0.002	W=0.956, P=0.004

Supplementary Table 2. Proportional odds assumption testing (Brant test) and model fit (AIC) for ordinal logistic regression models

**Model**	**χ^2**	***P***	**AIC**
Model 1 – TMT	1.63	0.20	519.45
Model 2 – TMT	4.26	0.23	519.32
Model 3 – TMT	6.10	0.53	522.48
Model 1 – MMT	0.51	0.47	529.78
Model 2 – MMT	2.42	0.49	531.78
Model 3 – MMT	4.76	0.69	536.22

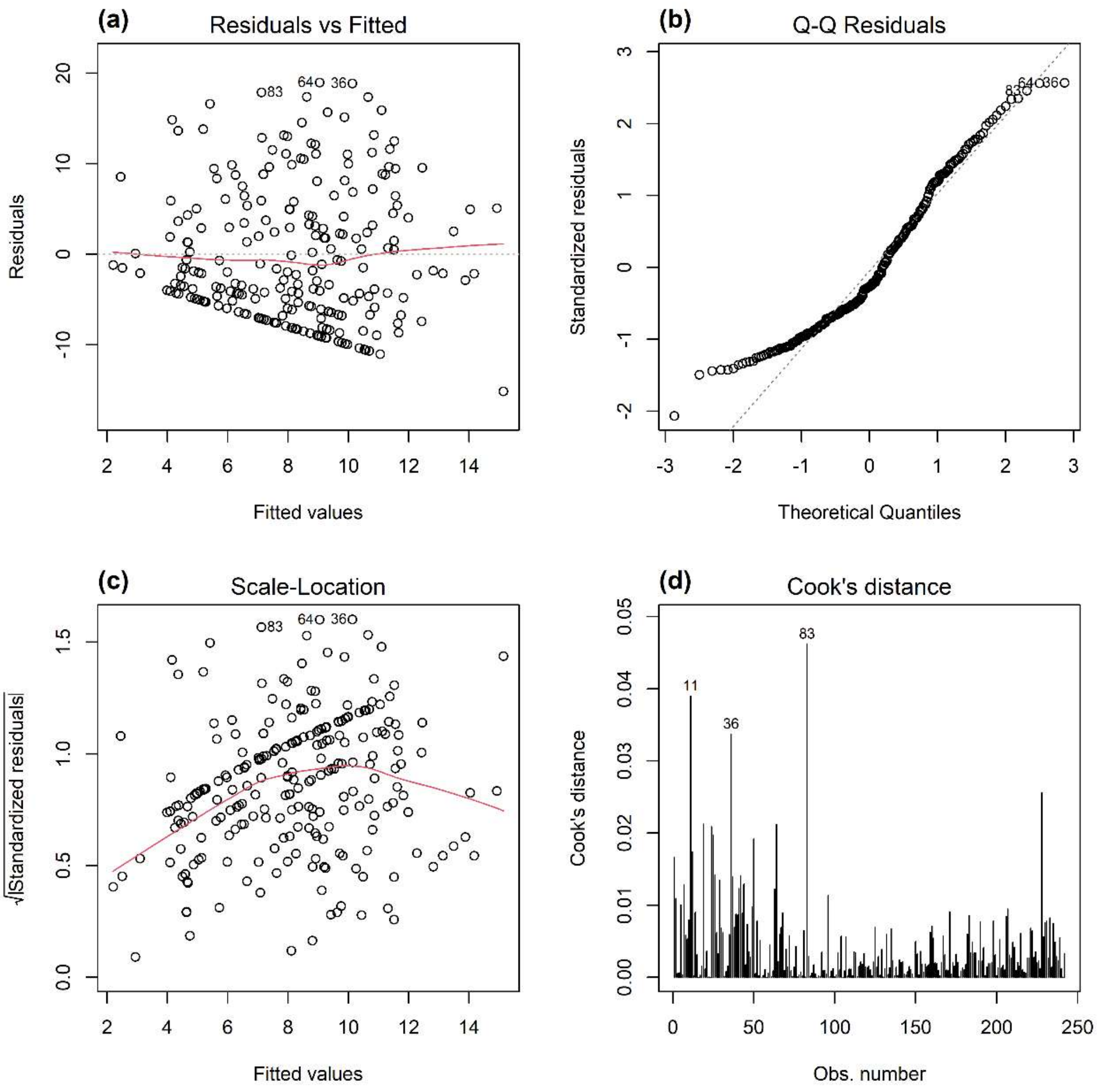

Supplementary Figure 1. Residual diagnostic plots for the multiple linear regression model with TMT

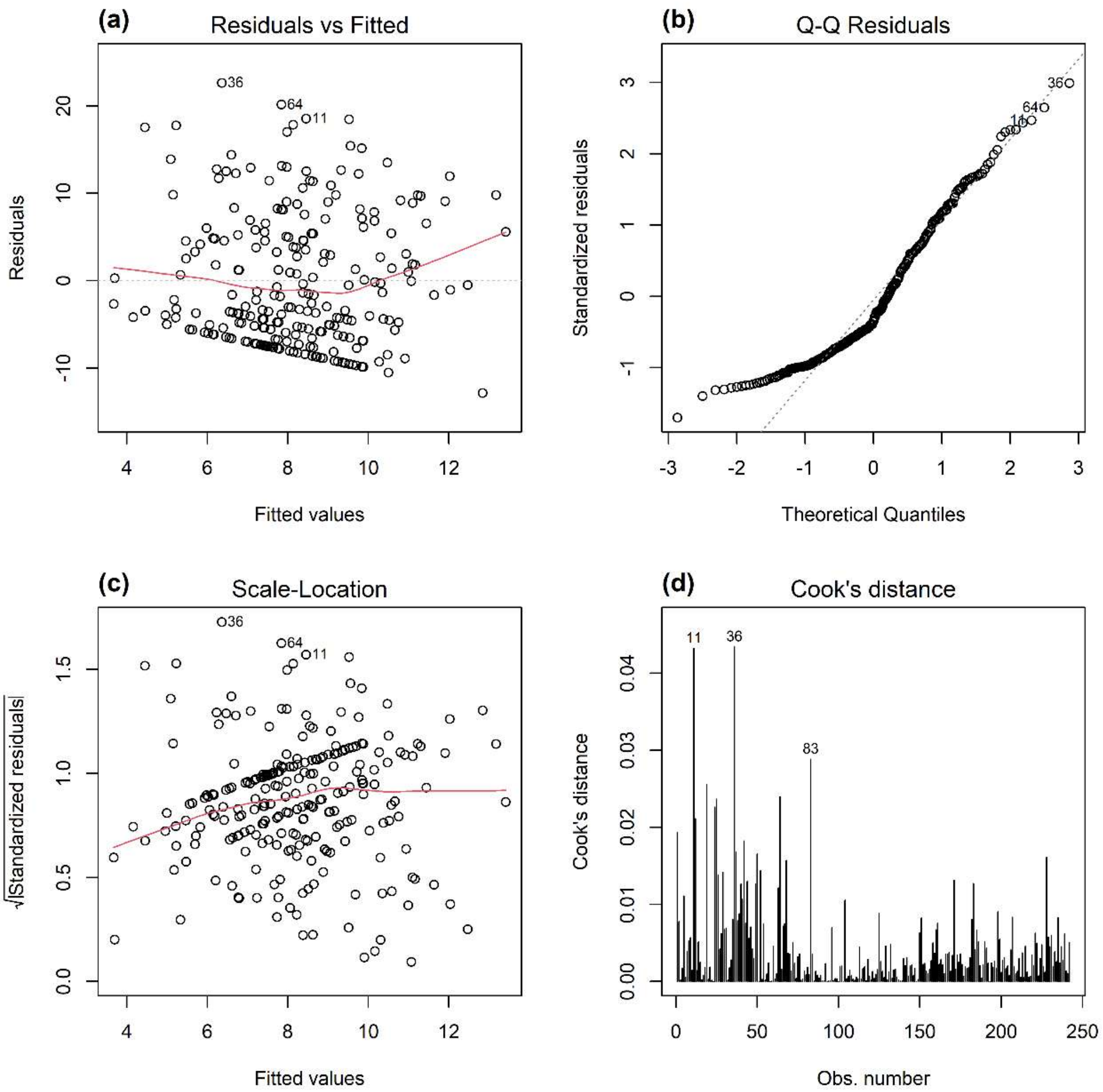

Supplementary Figure 2. Residual diagnostic plots for the multiple linear regression model with MMT

Supplementary Table 3. Sensitivity analysis using HC3 heteroskedasticity-consistent standard errors for multiple linear regression models

**Exposure**	**Variable**	**Beta**	**Robust SE**	**t**	***P***
TMT	Intercept	-24.736	8.899	-2.78	0.006
TMT	TMT, per 1 mm	3.074	0.741	4.15	<0.001
TMT	Age, years	0.123	0.074	1.66	0.098
TMT	Male sex	-1.375	1.874	-0.73	0.464
TMT	Education, years	0.215	0.117	1.83	0.068
TMT	Family history, yes	1.317	1.204	1.09	0.275
TMT	APOE ε4 positive	-1.358	0.989	-1.37	0.171
TMT	ASMI, kg/m²	-0.045	0.842	-0.05	0.957
MMT	Intercept	-11.092	8.840	-1.25	0.211
MMT	MMT, per 1 mm	0.505	0.288	1.76	0.080
MMT	Age, years	0.131	0.078	1.68	0.095
MMT	Male sex	-0.524	1.981	-0.26	0.792
MMT	Education, years	0.235	0.120	1.97	0.051
MMT	Family history, yes	1.132	1.283	0.88	0.378
MMT	APOE ε4 positive	-1.283	1.025	-1.25	0.212
MMT	ASMI, kg/m²	0.252	0.918	0.27	0.784
